# An approach to control of bioreactors. Application of the gain–scheduling method

**DOI:** 10.1155/S1463924699000073

**Published:** 1999

**Authors:** Estrella Alvarez, Carmen Riverol, J. M. Correa, J. M. Navaza

**Affiliations:** 1 Department of Chemical Engineering University of Vigo Vigo 36200 Spain; 2 Department of Chemical Engineering University of Santiago de Compostela Santiago 15007 Spain

## Abstract

A control system based on a combination of the gain-scheduling control method and an adaptive PID controller was designed for the production of xylose from hardwood hemicellulose using a stirring tank reactor. Different operating conditions have been considered for estimating the adjustable parameter (θ) to take into account the changes of energy of the system. The performance of the control system was studied first by numerical simulation, and after implementation in the stirred tank reactor where the controller actually works.


Journal of Automated Methods & Management in Chemistry, Vol. 21, No. 2 (March–April 1999) pp. 39–43

### An approach to control of bioreactors. Application of the gain-scheduling method

**Estrella Alvarez, Carmen Riverol, J. M. Correa and J. M. Navaza**[1]

*Department of Chemical Engineering, University of Vigo, Vigo-36200, Spain;*
*[1] Department of Chemical Engineering, University of Santiago de Compostela, Santiago 15007, Spain*

*A control system based on a combination of the gain-scheduling control method and an adaptive PID controller was designed for the production of xylose from hardwood hemicellulose using a stirring tank reactor. Different operating conditions have been considered for estimating the adjustable parameter ($\theta$) to take into account the changes of energy of the system. The performance of the control system was studied first by numerical simulation, and after implementation in the stirred tank reactor where the controller actually works.*

### Introduction

Temperature control in xylose production has industrial interest due to the following factors.

- Temperature control in xylose production plays an important role in several industries, e.g. biomass, cellulose, paper and lignin.
- Economical conversion of wood requires efficient use of the reactor and processing conditions.
- The behaviour of real plants includes the effects of several elements, e.g. real stirred tanks with dead zones, flow short circuits, and instruments that can introduce noise and inaccuracy into the measurement, transmission and manipulation of process variables.

Although production of chemicals from hardwood hemicellulose has been considered broadly from a resource point of view [1], progress toward commercial processes requires detailed study and analysis on a case by case basis. It is necessary to consider specific chemical processes, assess the technology available to accomplish each process, and thus to determine the economic viability of each process. Optimal production of xylose from hardwood hemicellulose must be analysed with respect to reactor yield, production concentration and operation conditions. Determination of the most desirable processing conditions requires the ability to predict reactor performance from knowledge of hydrolysis kinetics and pertinent transport properties.

The aim of this work is to evaluate the performance of a control system, and validate experimentally the controller adaptation in systems with dead zones and delay time.

### Study case: Description of the chemical process

### Notation

| | |
|---|---|
| $a$ | interfacial area per unit volume |
| $A$ | jacket heat transfer area |
| $A_f$ and $A_s$ | fast and slowly reacting solid xylan |
| $b$ | half-thickness of wood slab |
| $B$ | xylose monomer |
| $C_{Af}, C_{As}$ | concentration of fast and slowly reacting solid xylan |
| $C_{Af0}, C_{As0}$ | initial concentration of fast and slowly reacting solid xylan |
| $C_B$ | concentration of xylose with wood pores |
| $C_{BL}$ | concentration of xylose in free liquid |
| $C_{pL}$ | heat capacity of free liquid per unit mass |
| $C_{pg}$ | Heat capacity of the jacket per unit mass (glycol) |
| $D_e$ | effective diffusivity of xylose wood |
| $e$ | error signal |
| $F_j$ | volumetric flow rate in the jacket |
| $h$ | heat transfer coefficient at wood-free liquid interface |
| $k_B, k_{BL}$ | rate constants for decomposition of xylose in wood pores and free liquid, respectively |
| $k_f, k_s$ | rate constants for hydrolysis of fast and slowly reacting xylan |
| $k_L$ | mass transfer coefficient for xylose |
| $k_0$ | rate constant for hydrolysis of soluble xylan oligomers |
| $L$ | reactor length |
| $M$ | acid concentration of entering free liquid |
| $O$ | soluble xylan oligomers |
| $R_{Af}, R_{As}$ | mass rate of xylan hydrolysis for fast and slowly reacting fraction |
| $R_B, R_{BL}$ | mass rate of xylose degradation in wood pores and free liquid, respectively |
| $t$ | time |
| $T$ | temperature within wood particle |
| $T_f, T_0$ | initial or entering temperature of free liquid and wood chips, respectively |
| $T_j$ | jacket temperature |
| $T_L$ | temperature of free liquid |
| $U$ | overall jacket heat transfer coefficient |
| $u$ | manipulated variable (control input) |
| $u_i$ | interstitial fluid velocity |
| $v$ | average velocity of wood |
| $V_j$ | jacket volume |
| $x$ | distance from bottom of reactor |
| $X_f, X_s$ | fraction of original fast and slowly reacting xylan remaining |
| $y$ | distance from centre of wood particle |
| $z$ | fraction of original xylan identified as fast-reacting form |

 © 1999 Taylor & Francis Ltd
http://www.tandf.co.uk/JNLS/auc.htm
http://www.taylorandfrancis.com/JNLS/auc.htm

*Greek*

$\alpha$ effective thermal diffusivity of wood particle

$\varepsilon$ reactor void fraction

$\theta$ adjustable parameter

$\rho_g$ density of glycol

$\rho_L$ density of free liquid

The formation and degradation of xylose within the wood particles in a paper factory is the study case. Maloney and Chapman [1] and Levespiel [2] have described the model, although this paper considers the reactor inside the cooler jacket of glycol used to maintain a constant temperature. The following equations describe the real process:

$$A_f \rightarrow O \rightarrow B \rightarrow D \qquad A_s \rightarrow O \rightarrow B \rightarrow D \tag{1}$$

where $A_f$, $A_s$, $O$, $B$ and $D$ indicate fast and slowly reacting xylan, solubilized xylan oligomers, monomeric xylose, and degradation products, respectively. The corresponding rate expression for paper-birch xylan hydrolysis in sulphuric acid was determined in a previous study [3, 4] and given by:

$$R_x = z \cdot k_f \cdot X_f + (1 - z) \cdot k_s \cdot X_s \tag{2}$$

where $R_x$ is the fractional xylan hydrolysis rate, $X_f$ and $X_s$ are the fraction of fast and slowly reacing xylan remaining in the solid residue $z = 0.7$, and $k_f$ and $k_s$ are calculated using the equation reported in Ref. [1]. The following assumptions were made in developing the reactor model.

(1) The kinetics of xylose formation and degradation follow the models presented earlier.
(2) Axial dispersion can be neglected.
(3) The size of wood particles is uniform.
(4) The macroscopic reactor void fraction and the porosity of the wood are both constant.

The energy equation for a wood particle simplifies to

$$\frac{\partial T}{\partial t} = \alpha \cdot \frac{\partial^2 T}{\partial y^2} + \text{heat reaction} \tag{3}$$

where $\alpha$ is the effective thermal diffusivity and the other terms are defined in the notation section. The boundary conditions are:

$$t = \frac{x}{u_i}$$
$$T = T_0 \quad \text{for} - b < y < b$$
$$y = 0 \quad \frac{\partial T}{\partial y} = 0 \tag{4}$$
$$y = b$$
$$-k \cdot \frac{\partial T}{\partial y} = h \cdot (T - T_L) \quad \text{for} \quad t > \frac{x}{u_i}$$

where $k$ is the thermal conductivity of the composite and $T_L$ is the temperature of free liquid surrounding the particles. The energy equation for the liquid is:

$$\varepsilon \cdot \rho_L \cdot C_{pL} \frac{\partial T_L}{\partial t} = -\varepsilon \cdot u_i \cdot \rho_L \cdot C_{pL} \frac{\partial T_L}{\partial x} + a \cdot h(T_{y=b} - T_L) \tag{5}$$

where $\varepsilon$ is the reactor void fraction, $\rho_L$ the density of the liquid, $C_{pL}$ the heat capacity, $a$ the interfacial area per unit reactor volume, and $h$ the heat-transfer coefficient. The boundary conditions to be met are:

$$x = 0 \quad T_L = T_f$$
$$\varepsilon \cdot u_i \cdot \rho_L \cdot C_{pL} \frac{\partial T_L}{\partial x} = a \cdot h(T_0 - T_L) \quad \text{for} \quad t = \frac{x}{u_i} \tag{6}$$

The conservation equations for the xylan fractions within the solid are:

$$R_{A-\text{slow}} = k_s C_{AS} \qquad R_{A-\text{fast}} = k_s C_{Af} \tag{7}$$

with boundary conditions

$$t = \frac{x}{u_i} \quad C_{As} = C_{As0}$$
$$t = \frac{x}{u_i} \quad C_{Af} = C_{Af0}$$

For xylose in wood pores, the conservation equation is

$$\frac{\partial C_B}{\partial t} = \frac{D_e}{\theta} \frac{\partial^2 C_B}{\partial y^2} + R_{As} = R_{Af} - R_B$$

The boundary conditions for this system are:

$$t = \frac{x}{u_i} \quad C_B = 0$$
$$y = 0 \quad \frac{\partial C_B}{\partial y} = 0$$
$$y = b - D_e \frac{\partial C_B}{\partial y} = k_L(C_B = C_{BL})$$

For xylose in the liquid, the conservation law is:

$$u_i \cdot \varepsilon \cdot \frac{\partial C_{BL}}{\partial t} = -\varepsilon \cdot u_i \frac{\partial C_{BL}}{\partial x} + a \cdot k_L(C_{B(y=b)} - C_{BL}) - \varepsilon \cdot R_{BL} \tag{8}$$

The appropriate boundary conditions are:

$$x = 0, \quad C_{BL} = 0$$
$$t = \frac{x}{v}, \quad C_{BL} = 0$$

The energy equations and mass conservation equations are not coupled, the energy equations can be solved first by discretizing the variables in the space using the finite difference method. The procedure consists of alternatively solving the energy and mass balance equations, and continues until the desired reaction time is reached.

As well as the energy balances in the reactor, an additional equation exists that is the energy balance in the glycol jacket for the maintained constant temperature. The expression is as follows [5]:

*Table 1. Data of the reactor.*

| *Reactor type* | *Counter-current* |
|---|---|
| Entering fluid temperature (°C) | 242 |
| Reactor operating temperature (°C) | 156 |
| Wood residence time (min) | 58 |
| Liquid residence time (min) | 62 |

$$\frac{\partial T_j}{\partial t} = -\frac{F_j}{V_j} \cdot \frac{\partial T_j}{\partial x} + \frac{U \cdot A}{\rho_g \cdot C_{pg} \cdot V_j}(T_L - T_j) \qquad (9)$$

The data used in all the equations are shown in table 1. This model concludes that the control model for the temperature is third order [the three energy balances include solid (wood), free liquid and jacket]. Firstly, the bio-reactor was studied numerically to obtained information about the behaviour of the system, examine the transition from one region to another of stability, and the time response. Moreover, the gain-scheduling method requires the testing of different control algorithms, choosing the most efficient based on some operating conditions, therefore, the use of the model and the numerical simulation permitted the easiest design of control algorithms.

### Experimental set-up

The experimental set-up is shown in figure 1. The reactor is a continuous stirred tank with a wood residence time of 58 min. The runs are carried out at room temperature (around 25°C). An intelligent transmitter of temperature (Honeywell) with an accuracy of 0.15°C for Pt-100 (temperature sensor made of platinum alloy), output signal of 4–20 mA, and digital protocols DE or HART, was used in the temperature measurement. In total, four Pt-100 were used, one in each side of the tank. Moreover, the device has a weir that provides a constant volume of reacting mixture in the tank. When the Pt-100 is inside this device, there is a delay time that depends on the total flow rate of the liquid. The delay was measured experimentally from the temperature–response curve obtained by feeding the reactor with wood and injecting steps of sulphuric acid. The delay ranges of the Pt-100 work inside the range of flows is between 1 and 1.5 s, showing that we can neglect the delay time. The control loop includes the on-line implementation of the algorithm involved in the control model (gain-scheduling). The transmitter signal goes to the computer through an A/D interface (ADC, board model 2811, 2.5 V). The computer code was implemented in Visual Basic 5.0 pro (Microsoft-Spain, version in Spanish) in a PC-486 computer. The time required to compute the algorithm is approximately 2.2 s. The sampling time was set at $\Delta t = 3$ s. The computer manages the digital input and normalization of the temperature and output signal transmitted to the valve. The output signal is converted by an A/D interface. The valve is a pneumatic control valve (Badger Meter model D-research, coefficient 0.8) fitted with an I/P converter (Bellofram 1000 input 4–20 mA output 3–15 psi).

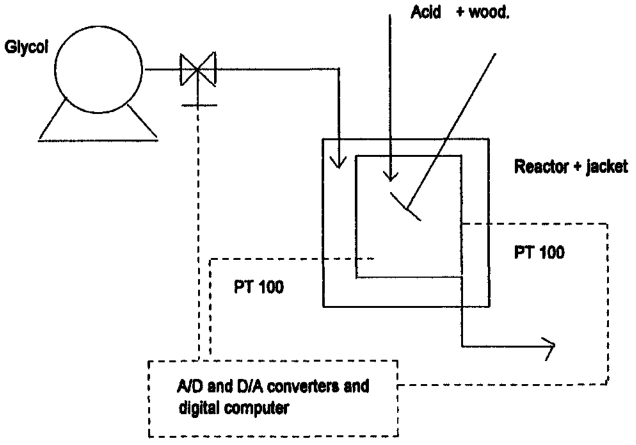

*Figure 1. Diagram of the process.*

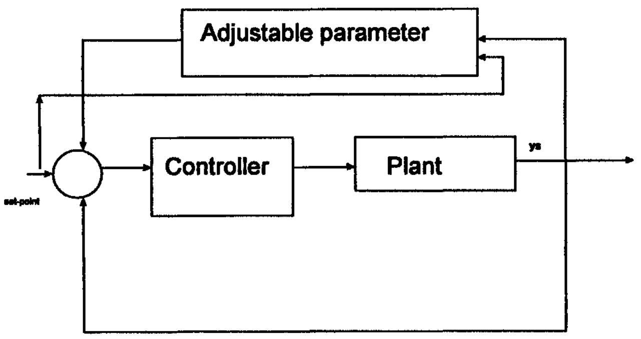

*Figure 2. Gain-scheduling diagram.*

Gain-sacheduling (figure 2) is a process by which one of several different control algorithms is chosen based on some operating conditions [6]. This process is thus adaptive because the control algorithms are designed off-line with *a priori* information, the main burden of gain-scheduling is identifying the proper control design to be used and effecting a smooth transfer from one design to another during system operation. Effectively, the design is broken into regions of operation, and in each region a fixed control design is used. The simulation (system model) is used to examine the transition from one region to another. It should be noted that often the question of stability is circumvented to having very few regions and ensuring that the controller will pass from one to another without getting trapped at the boundary. It is reasoned that for few regions that are switched very infrequently, or for a continuous set of parameters that are modified very slowly, the system stability can be ensured by examining each of the linear control laws as if it were fixed. Thus, gain-scheduling is usually thought of as dependent on some slowly varying parameter. In practice, stability analysis of gain-scheduled systems consists of close examination of system simulations [7]. Although gain-scheduling is adaptive, it is perhaps the least sophisticated of the strategies of adaptive control. This is because a relatively small number of designs are performed off-line and loaded into the controller as options that are subsequently chosen during the operation as a function of some measured or computed condtion. To be fair, gain-scheduling is also the most widely used of all adaptive algorithms by far, and it can be very effective.

### Discussion and results

In recent years, there has been extensive interest in adaptive control systems that automatically adjust the controller settings to compensate for unanticipated changes in the process or environment. Now we would like to offer a controller which is typically adaptive for temperature control in chemical processes. It is a problem control where process changes cannot be anticipated or inferred from process measurements. The design strategy is as follows.

First, the controller is proposed to have a similar structure to the PID, the expression as follows:

$$u(t) = \theta \cdot \left[e + \frac{1}{k_i}\int_0^t edt + k_d \cdot \frac{de}{dt}\right]$$

where $\theta$ is an adjustable parameter. In order to calculate the characteristic parameters of the controller, $k_i$ and $k_d$, the harmonic balance method of unit closed-loop was used [8], as reported in the literature. The proportional gain in the adaptive PID is fitted in one. If a setpoint of zero is assumed, a limit cycle can be considered with period $T_u$ and frequency $w_u$ such that the amplitude is $d$; by Fourier series the output in the first harmonic is $4d/\pi$, this means that the error signal has $\alpha$ amplitude of $\alpha = (4d/\pi)|G(jw_u)|$. The oscillation condition is such that:

$$\arg G(jw_u) = -\pi$$

$$K_u = \frac{4d}{\alpha\pi}$$

where $\alpha$ is the amplitude of the sine signal. The controller parameters are calculated in a similar manner to Ziegler Nichol's turning:

$$k_i = 0.5\, T_u$$

$$k_d = 0.12\, T_u$$

Next, the adjustable parameter $\theta$, that we proposed, is estimated using a macroscopic energy balance over the control zone (the control variable is the liquid temperature in the reactor) formed by the free liquid. Recall the energy balance of the liquid:

$$\varepsilon \cdot \rho_L \cdot C_{pL}\frac{\partial T_L}{\partial t} = -\varepsilon \cdot u_i \cdot \rho_L \cdot C_{pL}\frac{\partial T_L}{\partial x} + a \cdot h(T_{y=b} - T_L)$$

With the use of state feedback and coordinate transformations, a pole placement can be performed on the transformed linear system, and the resulting system will have the form [9] $\theta(\partial y/\partial t) + y = 0$ where $\theta$ is a pole placement parameter and $y$ the control variable, then

$$\theta\frac{\partial T_L}{\partial t} + T_L = 0 \quad (10)$$

Substituting equation (11) into equation (10) and assuming that $T_L$ has few changes along $x$, then

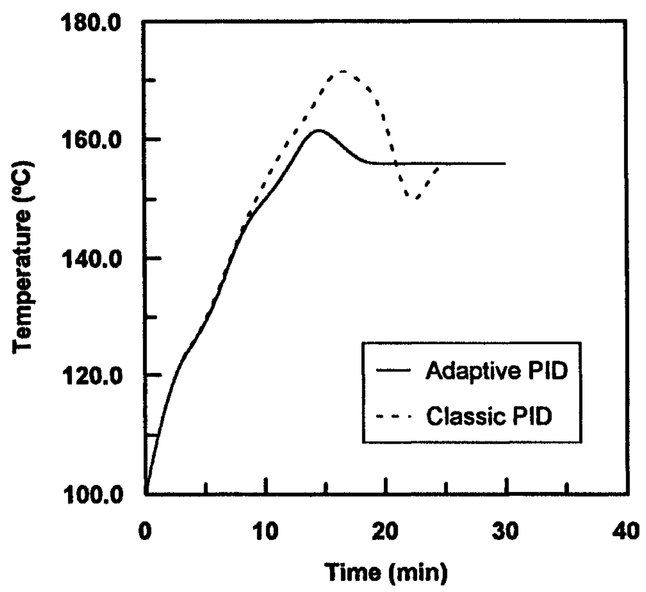

*Figure 3. Behaviour of the system with a step of −50%.*

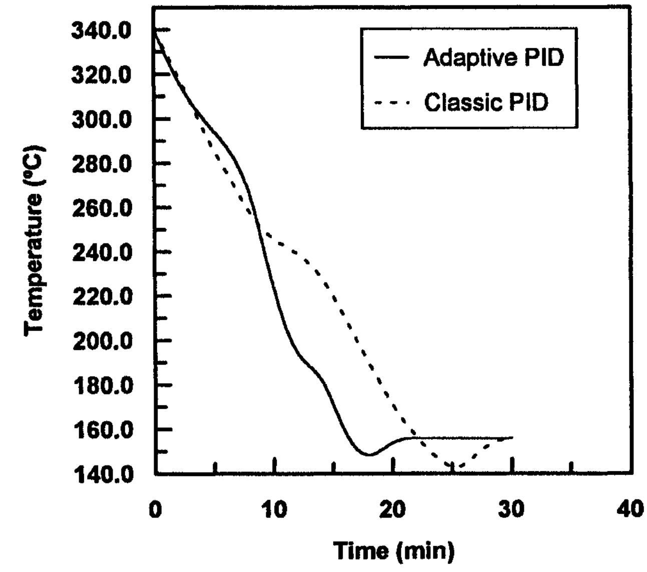

*Figure 4. Behaviour of the system with a step of 100%.*

$$\frac{u_i}{L}(T_L(L) - T_L(0)) - \frac{a \cdot h}{\varepsilon \cdot \rho_L \cdot C_{pL}} T_{y=b} + \left(\frac{a \cdot h}{\varepsilon \cdot \rho_L \cdot C_{pL}} - \frac{1}{\theta}\right) \cdot T_L = 0 \quad (11)$$

The parameter $\theta$ is calculated using equation (11), and $1/\theta$ indicates a frequency of changes of the pole placement (unit is $s^{-1}$). This adjustable parameter facilitates the process operation over a wide range of conditions. Using the reactor in different conditions, considering that the manipulated variable is the glycol flow and the setpoint is equal to 156°C, the results are shown in table 2, and figures 3 and 4. In table 2, stabilization time is depicted as shorter than the classic PID. This is a consequence of the fact that in equation (11) $\theta$ depends approximately on $T_L$ in the extremes (the temperature in the centre and the wall of the reactor). If the temperature

*Table 2. Behaviour of the system to different entering temperatures.*

| Entering fluid temperature (°C) | Reaction time + stabilization time (min) with adaptive PID | Reaction time + stabilization time (min) with classic PID | Adjustable parameter ($\theta$) |
|---|---|---|---|
| 230 | 20 | 29 | 2 |
| 90 | 22.5 | 33.2 | 3 |
| 100 | 18.5 | 25.3 | 2.66 |
| 310 | 20.3 | 27.8 | 4 |
| 340 | 19 | 27.9 | 4 |
| 200 | 23 | 28 | 3.1 |
| 330 | 25 | 33.2 | 4.8 |

difference is low, PID adaptive with respect to PID classic changes slightly, but if the difference is large then the controllers have a totally different behaviour [10, 11]. In figures 3 and 4, the behaviours of the system using a step of −50% and a step of 100% in the feed temperature are shown. The adaptive controller yields a small peak with respect to the classic PID, and the stabilization time is shortest in the adaptive compared to the classic PID. These results permit the verification of the adaptive capacity of the controller with respect to different input conditions.

### Conclusions

The applicability of a PID controller based on an adjustable parameter that depends on the temperature was discussed and illustrated with experiments involving different types of entering temperature (operation condition) using the xylose production process. The experimental validation of the controller shows that the control effciency is good with several perturbations in the operation conditions, and the stabilization time and speed of response is better than the classical control method. The controller performance is good under these conditions, although we think it can have limitations if it is used in other types of systems (pH control, electrical process), therefore the calculation of $\theta$ must be rewritten.

### Acknowledgment

We wish to thank everyone who worked on this paper, in particular the Mampe factory for permitting the publication of the results, and the anonymous referees for the helpful comments.

### References

1. Maloney, M. and Chapman, W., 1986, *Biotechnology Process*, **4**, 198.

2. Levespiel, L., 1980, *Ingenieria de las Reacciones Quimicas*, 1st edn (Mexico: Revete).

3. Song, S. and Lee, Y., 1983, *Chemical Engineering Communication*, **17**, 23.

4. Thompson, D. and Grthlein, H., 1979, I. & E. C. Prod. Res. Dev., **18**, 166.

5. Bequette, B., 1998, *Process Dynamic*, 1st edn (NY: Prentice Hall).

6. Hang, C. and Parks, P., 1982, *IEEE T.A.C. Vol AC*, **18**, 1.

7. Seborg, D., Edgar, T. and Shah, S., 1986, *AIChE J.*, **32**, 881.

8. Schmidt, L., 1978, *Hewlett Packard Journal*, **29**, 5.

9. Oroskar, A. and Stern, S., 1979, *AIChE J.*, **25**, 261.

10. Gupta, A. and Thodos, G., 1962, *Chem. Engng Prog.*, **58**, 7.

11. Slotile, J. and Yu, W., 1991, *Applied Nonlinear Control*, 2nd edn (Prentice Hall).

